# Evaluating the Use of Genetics in Brugada Syndrome Risk Stratification

**DOI:** 10.3389/fcvm.2021.652027

**Published:** 2021-04-21

**Authors:** Michelle M. Monasky, Emanuele Micaglio, Emanuela T. Locati, Carlo Pappone

**Affiliations:** ^1^Arrhythmology Department, IRCCS Policlinico San Donato, Milan, Italy; ^2^Vita-Salute San Raffaele University, Milan, Italy

**Keywords:** Brugada syndrome, sudden cardiac death, genetic testing, mutation, variant, *SCN5A*, sodium channel, arrhythmia

## Abstract

The evolution of the current dogma surrounding Brugada syndrome (BrS) has led to a significant debate about the real usefulness of genetic testing in this syndrome. Since BrS is defined by a particular electrocardiogram (ECG) pattern, after ruling out certain possible causes, this disease has come to be defined more for what it is *not* than for what it *is*. Extensive research is required to understand the effects of specific individual variants, including modifiers, rather than necessarily grouping together, for example, “all *SCN5A* variants” when trying to determine genotype-phenotype relationships, because not all variants within a particular gene act similarly. Genetic testing, including whole exome or whole genome testing, and family segregation analysis should always be performed when possible, as this is necessary to advance our understanding of the genetics of this condition. All considered, BrS should no longer be considered a pure autosomal dominant disorder, but an oligogenic condition. Less common patterns of inheritance, such as recessive, X–linked, or mitochondrial may exist. Genetic testing, in our opinion, should not be used for diagnostic purposes. However, variants in *SCN5A* can have a prognostic value. Patients should be diagnosed and treated per the current guidelines, after an arrhythmologic examination, based on the presence of the specific BrS ECG pattern. The genotype characterization should come in a second stage, particularly in order to guide the familial diagnostic work-up. In families in which an *SCN5A* pathogenic variant is found, genetic testing could possibly contribute to the prognostic risk stratification.

## Introduction

**Figure 1:**
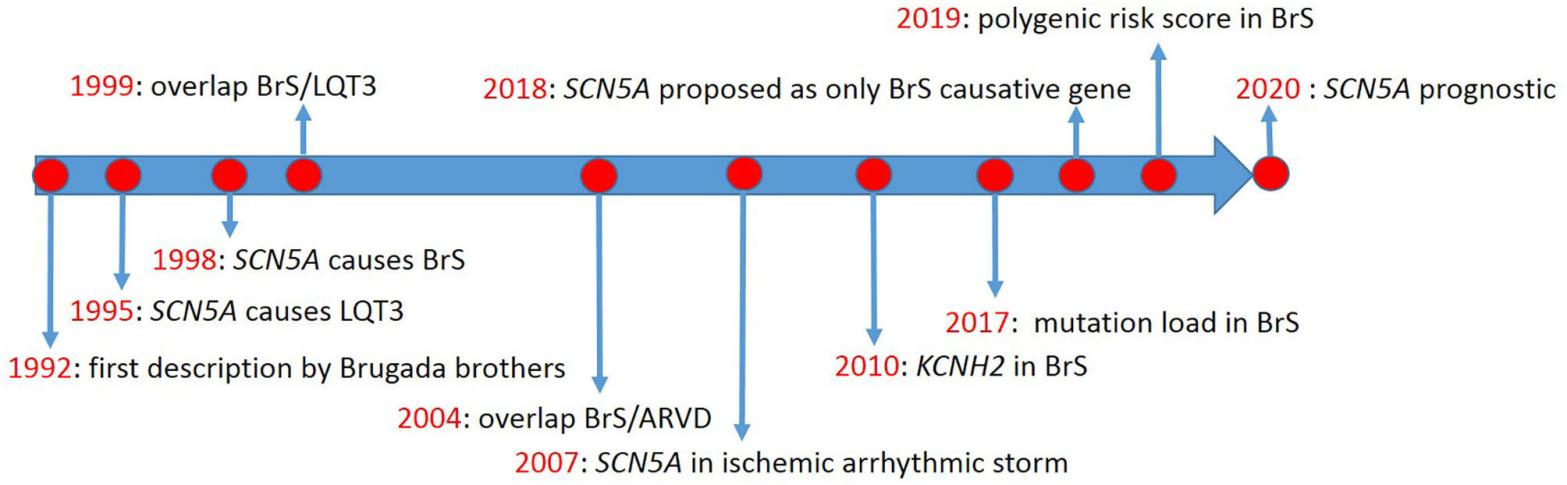
Timeline of Brugada syndrome discoveries.

The first description of Brugada syndrome (BrS) included eight unrelated patients with recurrent aborted sudden cardiac death due to ventricular fibrillation (VF) (1), in whom basal ECG showed persistent ST-segment elevation in precordial leads V1 to V2-V3. However, the genetic background was not discussed. Thus, no genotype-phenotype relationship was established. Meanwhile, Gellens and coworkers characterized *SCN5A* for the first time (2). Later, *SCN5A* was described in two unrelated families with long QT syndrome (LQTS) type-3 (LQT3) (3) (timeline, Figure 1).

BrS was first considered a form of idiopathic VF, resulting from abnormal electrophysiologic activity in right ventricular epicardium (4). It was described to lie on the same spectrum of cardiac electrophysiologic pathology as LQT3, caused by the same variant in *SCN5A* (5). Today, BrS is considered a Mendelian disorder inherited in an autosomal dominant fashion, even if alternative mechanisms of inheritance have been recently proposed (6). In BrS patients, variants in *SCN5A* are found more commonly than in any other gene (7) but confirm the clinical diagnosis in only a minority of cases (8). Many other genes have been proposed to cause BrS, but their roles are hotly debated (9, 10), with some groups suggesting that only *SCN5A* should be used in BrS genetic testing (9). However, variants in *SCN5A* have long been known to not necessarily segregate with BrS (4, 11). Recently, patients harboring *SCN5A* variants were demonstrated to have a worse prognosis (12).

These challenges have resulted in two important consequences: an overestimation of *SCN5A* diagnostic value and a contemporary underestimation of the clinical significance of genes different from *SCN5A*. All considered, the goals herein are to reevaluate the clinical significance of genetic data found in patients with BrS and to provide new insights about the complex genetics of BrS.

### Clinical Definition of BrS

The difficulty in understanding BrS genetics may lie in the definition of BrS, based on the electrocardiogram (ECG), specifically the type-1 BrS pattern, an ST-segment elevation with coved morphology, ≥2 mm, often associated with a sharp transition from elevated ST-segment to negative T-wave, among right precordial leads V1-V2, positioned in the 2nd, 3rd, or 4th intercostal space (13). This type-1 BrS pattern can occur either spontaneously or be unmasked with intravenous administration of Class 1c antiarrhythmic drugs, such as ajmaline or flecainide (13). Recently, it was hypothesized that BrS might actually be a heterogeneous disease with a common ECG phenotype (14). While this phenotype has been commonly attributed to loss-of-function of the Na_V_1.5 cardiac sodium channel, such phenotype could result from a number of molecular origins, not only *SCN5A* variants, but also alterations in proteins that modify the channel, or even environmental influences. Regarding the environmental influences, “true BrS” is diagnosed by ruling out such causes as electrolyte disturbances or myocardial ischemia. BrS patterns in these cases are said to be “BrS phenocopies” (15, 16). We disagree with the definition of “phenocopy,” because it is based upon what BrS is *not* rather than providing a clear picture of what BrS *is*. This is especially concerning since environmental influences can have a pivotal role in BrS (17). Perhaps a better view would be to consider the “BrS pattern” as a warning of risk for sudden cardiac death, regardless of the underlying cause (18). We are aware that this concept challenges the autosomal dominant model of BrS, largely based on the accepted etiologic role of *SCN5A*.

**Figure 2:**
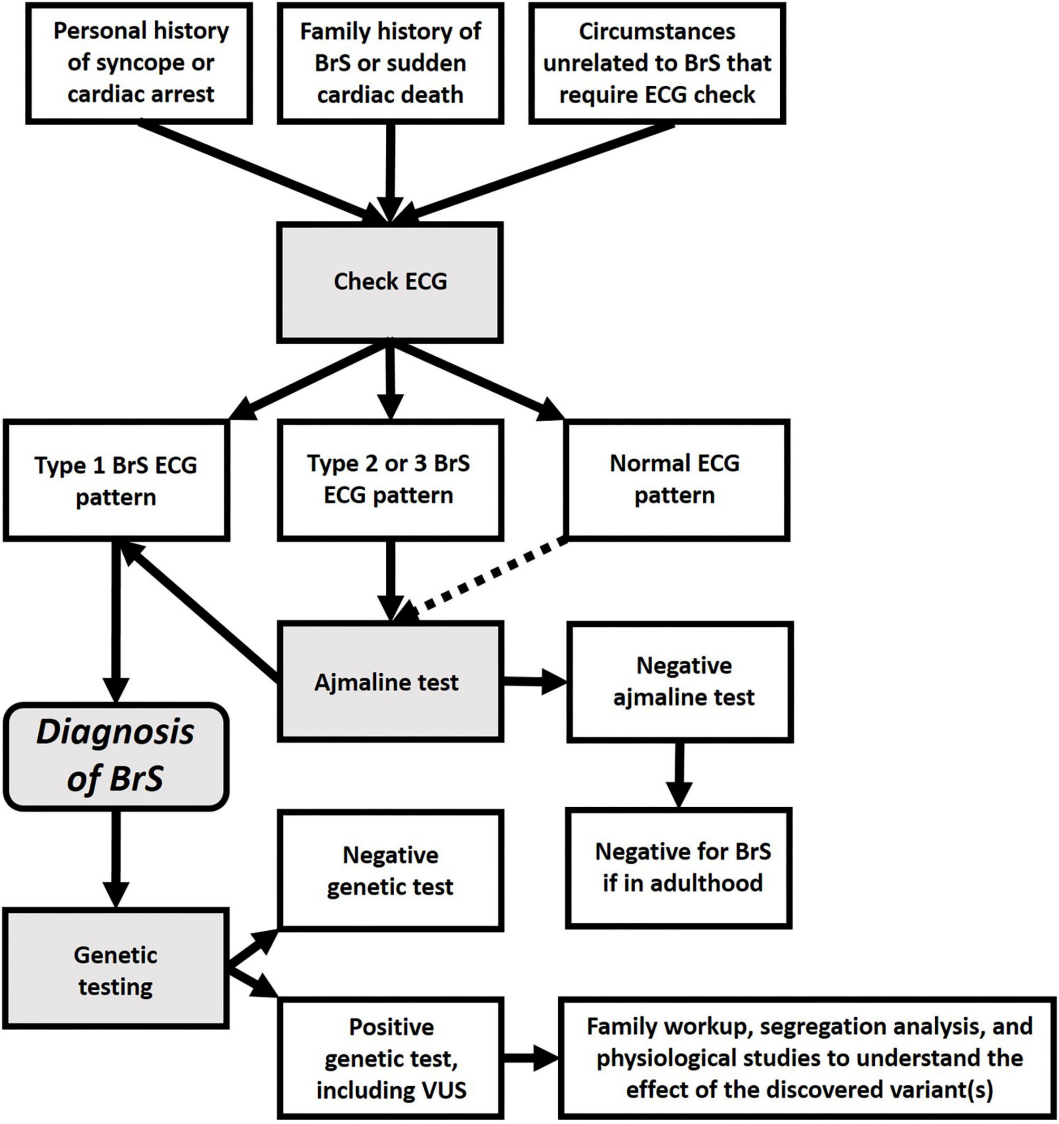
Flowchart for the diagnosis of BrS and genetic testing.

BrS has also been attributed to an increase in potassium current (19, 20). Furthermore, several studies have suggested BrS may be similar to a cardiomyopathy (21–26). Thus, it is likely that the ECG pattern used to define “BrS” is actually a common clinical manifestation, resulting from a multitude of different molecular causes. Further development of this concept may lead to a new paradigm for BrS, which may be considered not only as a Mendelian disorder, but as a complex condition, which might be caused by a huge variety of genetic variants, interacting with environmental factors (14, 27). In any case, since our current understanding of BrS genetics is still elementary, today BrS should be diagnosed by the type-1 ECG pattern (see Figure 2), not by genetic findings, especially additional findings during screening for other diseases.

### Genotype-Phenotype Relationships

Genotype-phenotype relationships are difficult to establish in BrS patients, because the clinical manifestations can be very subtle, and because the differential diagnosis can be extremely complex (28). Additionally, *SCN5A* variants have since been associated with a variety of pathologies (29, 30). Other works (31, 32) demonstrated both rare mutations and common variants in *SCN5A* can be considered phenotype modulators in myocardial infarction (33), arrhythmic storm (34), epilepsy (35), and even colon (36) and breast cancer (37). Thus, although *SCN5A* is the only undisputed gene in which mutations are thought to cause BrS, genetic testing alone is insufficient to diagnose BrS, as mutations in this gene could result in a number of different phenotypes. Instead, BrS must be diagnosed only in the presence of a diagnostic type 1 BrS ECG pattern (spontaneous or drug-induced), not due to secondary causes, such as electrolyte disturbances or myocardial ischemia.

### Other Candidate Genes

A recent study (9) concluded that only the *SCN5A* gene should be analyzed in BrS patients. We agree that mutations in *SCN5A* could be the cause of BrS in some patients. However, the study did not address what should be done in the majority of BrS patients, who test negative for any *SCN5A* mutations, nor provide clarity of the disease mechanism in those patients negative for *SCN5A* mutations, especially regarding the role of copy number variations and mitochondrial DNA. London expressed his disagreement, arguing that eliminating other genes from testing panels could stifle scientific advancement (10). Wilde and Gollob (38), however, countered by arguing that undue harm from incorrect interpretation could result in a life-changing diagnosis, require intervention, create life-long anxiety, and impact asymptomatic family members. We believe that suspected candidate genes should be tested and studied so that we can better understand their effects. However, all suspected cases should be confirmed by the presence of the BrS pattern, including patients found to have mutations in *SCN5A*, as single mutations in this gene are responsible for a variety of phenotypes, not only BrS (39, 40), and may not even cause BrS on their own (41).

Many other genes such as *SCN10A* (42, 43), *SCN4A* (44), *SCN1B* (45), *KCNH2*(46), *RANGRF* (47), *PKP2*(48), *TPM1*(49), and several calcium channels genes (50–53) have been described in patients clinically affected by BrS. Whole exome sequencing with a high coverage was performed in a family with both hypertrophic cardiomyopathy and type-1 BrS, apparently caused by the same heterozygous *TPM1* mutation (49). Thus, several candidate genes exist and should be further studied. Physiologic studies should follow the discovery of candidate mutations in the clinic, as abnormal effects in the physiology laboratory can provide useful insights to understanding particular new mutations.

### Modes of Inheritance

In spite of recent developments in the field of genetics, BrS is often still considered a monogenic Mendelian disease (54) inherited in an autosomal dominant fashion with incomplete penetrance (55–58). This is mainly due to the description of BrS in a family in which the genetics were consistent with this kind of transmission (59), making *SCN5A* the only accepted BrS gene (9). Another reason why *SCN5A* is so “popular” is because the segregation of variants in this gene show incomplete penetrance and marked variability in a significant percentage of patients (60). However, increasing evidence suggests that BrS in some patients might be actually caused by a digenic inheritance (61) or a combined effect of multiple variants (62), including polymorphisms (63). In this subset of patients, it is difficult to identify the real molecular cause of BrS, making it difficult to understand, using only genetic testing, which family members have inherited the syndrome and which have not. Additionally, since BrS may be due to a combined effect of multiple variants, the severity can often be different between family members (40). Furthermore, there might be other cases in which some family members have the syndrome but others do not, despite sharing certain variants, because of differences in modifier genes.

Although autosomal dominant inheritance with incomplete penetrance is the most commonly accepted mode of transmission of BrS, other forms of transmission have been suggested, such as recessive (64) and X-linked (19, 20). It is also possible that yet-undiscovered somatic mutations could have an effect on the heart. Furthermore, an autosomal dominant inheritance pattern could imply that the disease is Mendelian in nature, caused by a single mutation in a single gene. However, several studies have demonstrated an oligogenic mode of inheritance (7). Therefore, likely, in some families, a particular variant causes BrS in a Mendelian fashion, while in other families, the pattern of inheritance is more complicated to understand, because the disease is caused by a combination of factors, resulting in different phenotypes even between family members (65). Tadros et al. calculating polygenic risk scores (PRSs) for PR interval, QRS duration, and BrS, reported that 44 common variants associated with PR, and 26 common variants associated with QRS, in the general population, were associated with ajmaline-induced PR and QRS prolongation, respectively. Also, a 3-single-nucleotide-polymorphism PRS derived from a case-control BrS GWAS was independently associated with ajmaline-induced type-1 BrS ECG (66). This demonstrates the importance of polymorphisms that might predispose to arrhythmias and create a pathological effect, especially in the presence of other variants in the same patient.

### Overlap Syndromes

Since variants in *SCN5A* can be found in several cardiogenetic disorders, it is not surprising to observe an overlap between BrS and other pathologies. For example, BrS can be diagnosed in the proband while LQTS, epilepsy, febrile seizures, or complete bundle branch block can be present in the family members (67–70).

Overlap between arrhythmogenic right ventricular (RV) dysplasia/cardiomyopathy (ARVD/C) and BrS has been described by many groups (71), the mechanism of which may involve cell-cell junctions (24). Both ARVC and BrS can originate from mutations in the connexome, and the phenotype that emerges depends on the type of connexome mutation (72, 73). *PKP2* may be an important gene in this regard, as mutations in *PKP2* can result in loss of desmosomal integrity, cause sodium current deficit, and be found in patients with BrS (74, 75). The presence of ARVC in BrS patients has been associated with higher arrhythmic risk (76). The genetics of families with overlap syndromes should be carefully considered, as these genetic causes may be different than other families in which BrS is the only phenotype observed. This is yet another example of the need for personalized medicine and to consider the genetics of BrS on a family-by-family basis.

### Mitochondrial Considerations

Many recent studies have related cardiac arrhythmias, and particularly BrS, to mitochondrial function, or the effect of mitochondrial products on the sodium channel. Heart arrhythmias can originate from pathophysiology of the mitochondria, which produce adenosine triphosphate, a compound required for normal ion channel function (77). Aiba et al. described a family with BrS and the *SCN5A* mutation R526H, which is a PKA consensus phosphorylation site and associated with reduced basal I_Na_ due to the inability of PKA to act on the sodium channel to increase the sodium current (78). A mutation in the GPD1-L protein reduces I_Na_ by raising intracellular NADH levels and inducing reactive oxygen species (ROS) (79). This process of ROS production, its release from mitochondria, and thus its detrimental effect on the sodium current can be reversed in several ways, namely by NAD+, inhibition of mitochondrial electron transport, a mitochondrial targeted antioxidant, and an inner membrane anion channel modulator (80). A specific mitochondrial DNA (mtDNA) allelic combination and a high number of mtDNA single nucleotide polymorphisms (SNPs) have been reported in association with more severe cases of BrS, suggesting that these are important cofactors in the expression of the clinical phenotype (81, 82). Tafti et al. suggested that BrS may be caused by mutations in mitochondrial transfer RNA (tRNA) genes, leading to deficiencies in the translational process of critical proteins of the respiratory chain (83). Reports have demonstrated that tRNAMet, tRNAIle, tRNATrp and tRNAGln genes are hot spots for cardiovascular diseases (83, 84). Thus, mitochondrial function, or malfunction, contributes to sodium channel function and to cardiac rhythm.

### Risk Stratification

Risk stratification in BrS has previously relied on clinical scores (85), including familial history of sudden cardiac death, personal history of syncope, aborted cardiac arrest, spontaneous type-1 BrS pattern, or male gender. It was also reported that proband status, inducibility toward ventricular arrhythmias (86), arrhythmogenic substrate area, and late potentials (87) were predictors of higher risk. Our group recently proposed the *SCN5A* genetic status as a prognostic factor for BrS patients (12, 88). In particular, *SCN5A* mutation carriers exhibited more pronounced epicardial electrical abnormalities and a more aggressive clinical presentation. In at least a subgroup of patients, the mutated *SCN5A* gene acts more like a phenotype modulator than a real Mendelian dominant cause of the displayed phenotype, possibly calling into question the autosomal dominant inheritance of BrS. This is true also for variants of “unknown significance” (VUS), which are generally treated as “benign.” However, in our experience, several of these VUS are later reclassified as pathogenic. We believe that, in time, many other VUS, especially in the *SCN5A* gene, will be determined to be pathogenic, considering also that the oligogenic model is likely to be accepted in the near future.

## Discussion

The genetics of BrS have likely remained elusive because of how the disease has been considered only an autosomal dominant Mendelian disorder. However, when BrS is considered an oligogenic disorder, it may be possible to use genetics to predict the BrS phenotype. Besides direct modifications in the Na_V_1.5 protein, its function can be altered by many regulatory proteins like Hey2, Mog1, Gpd1-L, and others. According to us, studying the genes encoding those proteins is very important for the clinical management of BrS patients. Additionally, environmental factors might influence channel function through post-translational modifications. Even in families where *SCN5A* variants have been found, segregation analysis is not always consistent with autosomal dominant inheritance, demanding caution be used when interpreting genetic test results. Currently, it is necessary that all suspected cases of BrS are confirmed with ECG, using, when necessary, drug challenge to elicit the type-1 pattern. In other words, genetic testing alone should not be used for diagnostic purposes at this time, but rather, the patients should each fulfill the diagnostic criteria for BrS at an arrhythmologic examination, as per the current guidelines (89). However, in families in which a *SCN5A* pathogenic variant is found, genetic testing could possibly contribute to the prognostic risk stratification.

Ideally, whole exome or whole genome testing should be performed to both confirm candidate genes and identify new ones. Collecting family segregation is mandatory to understand whether a particular variant might be clinically relevant. Ideally, such data should then be deposited into international databases. The specific effects of distinct variants should be studied, rather than necessarily grouping together, for example, “all *SCN5A* variants” when trying to determine genotype-phenotype relationships, because not all variants within a particular gene act similarly.

Identifying variants involved in oligogenic cases of BrS is extremely complicated. For this, the effect of polymorphisms, which, on their own, are considered benign, should be considered, as they may act as modifiers in the presence of other variants. For example, two variants in a particular gene may exist, which, individually, result in a benign phenotype, as neither variant, on their own, significantly modifies the ultimate function of the resulting protein. However, if those two (or three, or more) variants occur together in the same person, together they could ultimately impair the function of the protein, altering the clinical picture. This “mutational load” is an important concept in BrS, explaining why the genetics of this disease have been so difficult to elucidate. However, to understand the effect of mutational load, or compound heterozygosity (i.e., two or more heterogeneous recessive alleles at a particular locus), extensive research studies should be performed, also identifying other genes responsible for BrS, besides *SCN5A*. Only then it will be possible to study these concepts of oligogenic inheritance in the majority of patients. Probably, whole genome or whole exome studies would be useful in determining the genes involved, along with family segregation analysis.

Finally, non-genomic DNA considerations should be mentioned, as post-translational modifications of the sodium channel could affect its function without any variants in the *SCN5A* gene. Studies should be expanded to better understand any possible role for mitochondrial involvement, including the analysis of mitochondrial genes, their products, and their functional effects on the cells. Environmental factors should also be studied, including anything to which families may be exposed, resulting in post-translational effects, especially when probands test negative for variants in all BrS candidate genes. Environmental factors could be mistaken as a genetic condition when several family members living in the same environment are affected.

## Data Availability Statement

The original contributions presented in the study are included in the article/supplementary material, further inquiries can be directed to the corresponding author/s.

## Author Contributions

MM and EM drafted the paper. EL and CP provided revisions and useful feedback. CP secured funding for the project. All authors approved the final version of the manuscript.

## Conflict of Interest

The authors declare that the research was conducted in the absence of any commercial or financial relationships that could be construed as a potential conflict of interest.

## References

[1] Brugada P Brugada J. Right bundle branch block, persistent ST segment elevation and sudden cardiac death: a distinct clinical and electrocardiographic syndrome. A multicenter report. J Am Coll Cardiol. (1992) 20:1391–6. 10.1016/0735-1097(92)90253-J 1309182

[2] Gellens ME George ALJr Chen LQ Chahine M Horn R Barchi RL. Primary structure and functional expression of the human cardiac tetrodotoxin-insensitive voltage-dependent sodium channel. Proc Natl Acad Sci U S A. (1992) 89:554–8. 10.1073/pnas.89.2.554 1309946 PMC48277

[3] Wang Q Shen J Splawski I Atkinson D Li Z Robinson JL. SCN5A mutations associated with an inherited cardiac arrhythmia, long QT syndrome. Cell. (1995) 80:805–11. 10.1016/0092-8674(95)90359-3 7889574

[4] Gussak I Antzelevitch C Bjerregaard P Towbin JA Chaitman BR. The Brugada syndrome: clinical, electrophysiologic and genetic aspects. J Am Coll Cardiol. (1999) 33:5–15. 10.1016/S0735-1097(98)00528-2 9935001

[5] Bezzina C Veldkamp MW Van Den Berg MP Postma AV Rook MB Viersma JW. A single Na(+) channel mutation causing both long-QT and Brugada syndromes. Circ Res. (1999) 85:1206–13. 10.1161/01.RES.85.12.1206 10590249

[6] Campuzano O Sarquella-Brugada G Cesar S Arbelo E Brugada J Brugada R. Update on genetic basis of brugada syndrome: monogenic, polygenic or oligogenic? Int J Mol Sci. (2020) 21:7155. 10.3390/ijms21197155 32998306 PMC7582739

[7] Monasky MM Micaglio E Ciconte G Pappone C. Brugada syndrome: oligogenic or mendelian disease? Int J Mol Sci. (2020) 21:1687. 10.3390/ijms21051687 32121523 PMC7084676

[8] Kapplinger JD Tester DJ Alders M Benito B Berthet M Brugada J. An international compendium of mutations in the SCN5A-encoded cardiac sodium channel in patients referred for Brugada syndrome genetic testing. Heart Rhythm. (2010) 7:33–46. 10.1016/j.hrthm.2009.09.069 20129283 PMC2822446

[9] Hosseini SM Kim R Udupa S Costain G Jobling R Liston E. Reappraisal of reported genes for sudden arrhythmic death. Circulation. (2018) 138:1195–205. 10.1161/CIRCULATIONAHA.118.035070 29959160 PMC6147087

[10] London B. Letter by london regarding article, “reappraisal of reported genes for sudden arrhythmic death: evidence-based evaluation of gene validity for Brugada syndrome”. Circulation. (2019) 139:1758–9. 10.1161/CIRCULATIONAHA.118.036889 30933610 PMC7467328

[11] Wijeyeratne YD Tanck MW Mizusawa Y Batchvarov V Barc J Crotti L. SCN5A mutation type and a genetic risk score associate variably with brugada syndrome phenotype in SCN5A families. Circ Genom Precis Med. (2020) 13:e002911. 10.1161/CIRCGEN.120.002911 33164571 PMC7748043

[12] Ciconte G Monasky MM Santinelli V Micaglio E Vicedomini G Anastasia L. Brugada syndrome genetics is associated with phenotype severity. Eur Heart J. (2020) 42:1082–90. 10.1093/eurheartj/ehaa942 33221895 PMC7955973

[13] Antzelevitch C Yan GX Ackerman MJ Borggrefe M Corrado D Guo J. J-Wave syndromes expert consensus conference report: emerging concepts and gaps in knowledge. J Arrhythm. (2016) 32:315–39. 10.1016/j.joa.2016.07.002 27761155 PMC5063270

[14] Gray B Semsarian C Sy RW. Brugada syndrome: a heterogeneous disease with a common ECG phenotype? J Cardiovasc Electrophysiol. (2014) 25:450–6. 10.1111/jce.12366 24405173

[15] Genaro NR Anselm DD Cervino N Estevez AO Perona C Villamil AM. Brugada phenocopy clinical reproducibility demonstrated by recurrent hypokalemia. Ann Noninvasive Electrocardiol. (2014) 19:387–90. 10.1111/anec.12101 24147860 PMC6932150

[16] Maheshwari A Von Wald L Krishnan B Benditt DG. Hyperkalemia-induced brugada phenocopy. JACC Clin Electrophysiol. (2017) 3:1058–9. 10.1016/j.jacep.2016.12.012 29759712

[17] Yap YG Behr ER Camm AJ. Drug-induced Brugada syndrome. Europace. (2009) 11:989–94. 10.1093/europace/eup114 19482855

[18] Locati ET Bagliani G Cecchi F Johny H Lunati M Pappone C. arrhythmias due to inherited and acquired abnormalities of ventricular repolarization. Card Electrophysiol Clin. (2019) 11:345–62. 10.1016/j.ccep.2019.02.009 31084855

[19] Ohno S Zankov DP Ding WG Itoh H Makiyama T Doi T. KCNE5 (KCNE1L) variants are novel modulators of Brugada syndrome and idiopathic ventricular fibrillation. Circ Arrhythm Electrophysiol. (2011) 4:352–61. 10.1161/CIRCEP.110.959619 21493962

[20] David JP Lisewski U Crump SM Jepps TA Bocksteins E Wilck N. Deletion in mice of X-linked, Brugada syndrome- and atrial fibrillation-associated Kcne5 augments ventricular KV currents and predisposes to ventricular arrhythmia. FASEB J. (2019) 33:2537–52. 10.1096/fj.201800502R 30289750 PMC6338634

[21] Peters S. Is Brugada syndrome a variant of arrhythmogenic cardiomyopathy? Int J Cardiol. (2015) 189:88–90. 10.1016/j.ijcard.2015.03.394 25889434

[22] Peters S. The history of Brugada syndrome—continuum with arrhythmogenic cardiomyopathy or lone disease? Int J Cardiol. (2016) 211:84–5. 10.1016/j.ijcard.2016.02.132 26982088

[23] Moncayo-Arlandi J Brugada R. Unmasking the molecular link between arrhythmogenic cardiomyopathy and Brugada syndrome. Nat Rev Cardiol. (2017) 14:744–56. 10.1038/nrcardio.2017.103 28703223

[24] Ben-Haim Y Asimaki A Behr ER. Brugada syndrome and arrhythmogenic cardiomyopathy: overlapping disorders of the connexome? Europace. (2020). 10.1093/europace/euaa277. [Epub ahead of print]. 33200179

[25] Pappone C Micaglio E Locati ET Monasky MM. The omics of channelopathies and cardiomyopathies: what we know and how they are useful. Eur Heart J. (2020) 22(Suppl L):L105–L109. 10.1093/eurheartj/suaa146 33654474 PMC7904073

[26] Pappone C Monasky MM Micaglio E Ciconte G. Right ventricular electromechanical abnormalities in Brugada syndrome: is this a cardiomyopathy? Eur Heart J. (2020) 22(Suppl E):E101–E104. 10.1093/eurheartj/suaa071 32523450 PMC7270919

[27] Di Domenico M Scumaci D Grasso S Gaspari M Curcio A Oliva A. Biomarker discovery by plasma proteomics in familial Brugada syndrome. Front Biosci. (2013) 18:564–71. 10.2741/4120 23276942

[28] Dendramis G. Brugada syndrome and Brugada phenocopy. The importance of a differential diagnosis. Int J Cardiol. (2016) 210:25–7. 10.1016/j.ijcard.2016.02.097 26922708

[29] Laurent G Saal S Amarouch MY Beziau DM Marsman RF Faivre L. Multifocal ectopic Purkinje-related premature contractions: a new SCN5A-related cardiac channelopathy. J Am Coll Cardiol. (2012) 60:144–56. 10.1016/j.jacc.2012.02.052 22766342

[30] Wilde AAM Amin AS. Clinical spectrum of SCN5A mutations: long QT syndrome, brugada syndrome, and cardiomyopathy. JACC Clin Electrophysiol. (2018) 4:569–79. 10.1016/j.jacep.2018.03.006 29798782

[31] Maury P Moreau A Hidden-Lucet F Leenhardt A Fressart V Berthet M. Novel SCN5A mutations in two families with “Brugada-like” ST elevation in the inferior leads and conduction disturbances. J Interv Card Electrophysiol. (2013) 37:131–40. 10.1007/s10840-013-9805-7 23612926

[32] Jabbari R Glinge C Jabbari J Risgaard B Winkel BG Terkelsen CJ. A common variant in SCN5A and the risk of ventricular fibrillation caused by first ST-segment elevation myocardial infarction. PLoS ONE. (2017) 12:e0170193. 10.1371/journal.pone.0170193 28085969 PMC5234807

[33] Oliva A Hu D Viskin S Carrier T Cordeiro JM Barajas-Martinez H. SCN5A mutation associated with acute myocardial infarction. Leg Med. (2009) 11(Suppl 1):S206–209. 10.1016/j.legalmed.2009.02.044 19345130 PMC2813686

[34] Hu D Viskin S Oliva A Carrier T Cordeiro JM Barajas-Martinez H. Novel mutation in the SCN5A gene associated with arrhythmic storm development during acute myocardial infarction. Heart Rhythm. (2007) 4:1072–80. 10.1016/j.hrthm.2007.03.040 17675083 PMC1978483

[35] Aurlien D Leren TP Tauboll E Gjerstad L. New SCN5A mutation in a SUDEP victim with idiopathic epilepsy. Seizure. (2009) 18:158–60. 10.1016/j.seizure.2008.07.008 18752973

[36] House CD Vaske CJ Schwartz AM Obias V Frank B Luu T. Voltage-gated Na+ channel SCN5A is a key regulator of a gene transcriptional network that controls colon cancer invasion. Cancer Res. (2010) 70:6957–67. 10.1158/0008-5472.CAN-10-1169 20651255 PMC2936697

[37] Luo Q Wu T Wu W Chen G Luo X Jiang L. The functional role of voltage-gated sodium channel Nav1.5 in metastatic breast cancer. Front Pharmacol. (2020) 11:1111. 10.3389/fphar.2020.01111 32792949 PMC7393602

[38] Wilde AAM Gollob MH. Response by wilde and gollob to letter regarding article, “reappraisal of reported genes for sudden arrhythmic death: evidence-based evaluation of gene validity for brugada syndrome”. Circulation. (2019) 139:1760–1. 10.1161/CIRCULATIONAHA.119.039065 30933625

[39] Yagihara N Watanabe H Barnett P Duboscq-Bidot L Thomas AC Yang P. Variants in the SCN5A promoter associated with various arrhythmia phenotypes. J Am Heart Assoc. (2016) 5:e003644. 10.1161/JAHA.116.003644 27625342 PMC5079027

[40] Cerrone M Remme CA Tadros R Bezzina CR Delmar M. Beyond the one gene-one disease paradigm: complex genetics and pleiotropy in inheritable cardiac disorders. Circulation. (2019) 140:595–610. 10.1161/CIRCULATIONAHA.118.035954 31403841 PMC6697136

[41] Daimi H Khelil AH Neji A Ben Hamda K Maaoui S Aranega A. Role of SCN5A coding and non-coding sequences in Brugada syndrome onset: what's behind the scenes? Biomed J. (2019) 42:252–60. 10.1016/j.bj.2019.03.003 31627867 PMC6818142

[42] Monasky MM Micaglio E Vicedomini G Locati ET Ciconte G Giannelli L. Comparable clinical characteristics in Brugada syndrome patients harboring SCN5A or novel SCN10A variants. Europace. (2019) 21:1550–8. 10.1093/europace/euz186 31292628

[43] Trujillo-Quintero JP Gutierrez-Agullo M Ochoa JP Martinez-Martinez JG De Una D Garcia-Fernandez A. Familial brugada syndrome associated with a complete deletion of the SCN5A and SCN10A genes. Rev Esp Cardiol. (2019) 72:176–8. 10.1016/j.rec.2017.12.021 29650450

[44] Cavalli M Fossati B Vitale R Brigonzi E Ricigliano VAG Saraceno L. Flecainide-induced brugada syndrome in a patient with skeletal muscle sodium channelopathy: a case report with critical therapeutical implications and review of the literature. Front Neurol. (2018) 9:385. 10.3389/fneur.2018.00385 29899727 PMC5988887

[45] Ricci MT Menegon S Vatrano S Mandrile G Cerrato N Carvalho P. SCN1B gene variants in Brugada Syndrome: a study of 145 SCN5A-negative patients. Sci Rep. (2014) 4:6470. 10.1038/srep06470 25253298 PMC5377327

[46] Wilders R Verkerk AO. Role of the R1135H KCNH2 mutation in Brugada syndrome. Int J Cardiol. (2010) 144:149–51. 10.1016/j.ijcard.2008.12.177 19174314

[47] Campuzano O Berne P Selga E Allegue C Iglesias A Brugada J. Brugada syndrome and p.E61X_RANGRF. Cardiol J. (2014) 21:121–127. 10.5603/CJ.a2013.0125 24142675

[48] Campuzano O Fernandez-Falgueras A Iglesias A Brugada R. Brugada Syndrome and PKP2: evidences and uncertainties. Int J Cardiol. (2016) 214:403–5. 10.1016/j.ijcard.2016.03.194 27085656

[49] Mango R Luchetti A Sangiuolo R Ferradini V Briglia N Giardina E. Next generation sequencing and linkage analysis for the molecular diagnosis of a novel overlapping syndrome characterized by hypertrophic cardiomyopathy and typical electrical instability of brugada syndrome. Circ J. (2016) 80:938–49. 10.1253/circj.CJ-15-0685 26960954

[50] Antzelevitch C Pollevick GD Cordeiro JM Casis O Sanguinetti MC Aizawa Y. Loss-of-function mutations in the cardiac calcium channel underlie a new clinical entity characterized by ST-segment elevation, short QT intervals, and sudden cardiac death. Circulation. (2007) 115:442–9. 10.1161/CIRCULATIONAHA.106.668392 17224476 PMC1952683

[51] Cordeiro JM Marieb M Pfeiffer R Calloe K Burashnikov E Antzelevitch C. Accelerated inactivation of the L-type calcium current due to a mutation in CACNB2b underlies Brugada syndrome. J Mol Cell Cardiol. (2009) 46:695–703. 10.1016/j.yjmcc.2009.01.014 19358333 PMC2668128

[52] Burashnikov E Pfeiffer R Barajas-Martinez H Delpon E Hu D Desai M. Mutations in the cardiac L-type calcium channel associated with inherited J-wave syndromes and sudden cardiac death. Heart Rhythm. (2010) 7:1872–82. 10.1016/j.hrthm.2010.08.026 20817017 PMC2999985

[53] Monasky MM Pappone C Piccoli M Ghiroldi A Micaglio E Anastasia L. Calcium in brugada syndrome: questions for future research. Front Physiol. (2018) 9:1088. 10.3389/fphys.2018.01088 30147658 PMC6095984

[54] Sattar Y Ullah W Zaidi SR Almas T Alraies MC. Brugada pattern type 2 diagnosis unmasked by aspiration pneumonia. Cureus. (2020) 12:e8331. 10.7759/cureus.8331 32617208 PMC7325341

[55] Chen Q Kirsch GE Zhang D Brugada R Brugada J Brugada P. Genetic basis and molecular mechanism for idiopathic ventricular fibrillation. Nature. (1998) 392:293–6. 10.1038/32675 9521325

[56] Priori SG Napolitano C Giordano U Collisani G Memmi M. Brugada syndrome and sudden cardiac death in children. Lancet. (2000) 355:808–9. 10.1016/S0140-6736(99)05277-0 10711933

[57] Nademanee K Veerakul G Chandanamattha P Chaothawee L Ariyachaipanich A Jirasirirojanakorn K. Prevention of ventricular fibrillation episodes in Brugada syndrome by catheter ablation over the anterior right ventricular outflow tract epicardium. Circulation. (2011) 123:1270–9. 10.1161/CIRCULATIONAHA.110.972612 21403098

[58] Lieve KV Wilde AA. Inherited ion channel diseases: a brief review. Europace. (2015) 17(Suppl 2):ii1–6. 10.1093/europace/euv105 26842110

[59] Brugada R Campuzano O Sarquella-Brugada G Brugada P Brugada J Hong K. Brugada Syndrome. In: Adam MP Ardinger HH Pagon RA Wallace SE Bean LJH Stephens K Amemiya A editors. GeneReviews® [Internet]. Seattle, WA: University of Washington, Seattle 1993–2020 (2005). 20301690

[60] Garcia-Molina E Sabater-Molina M Munoz C Ruiz-Espejo F Gimeno JR. An R1632C variant in the SCN5A gene causing Brugada syndrome. Mol Med Rep. (2016) 13:4677–80. 10.3892/mmr.2016.5100 27082542

[61] Gualandi F Zaraket F Malagu M Parmeggiani G Trabanelli C Fini S. Mutation load of multiple ion channel gene mutations in brugada syndrome. Cardiology. (2017) 137:256–60. 10.1159/000471792 28494446

[62] Wu Y Ai M Bardeesi ASA Xu L Zheng J Zheng D. Brugada syndrome: a fatal disease with complex genetic etiologies - still a long way to go. Forensic Sci Res. (2017) 2:115–25. 10.1080/20961790.2017.1333203 30483629 PMC6197104

[63] Makarawate P Glinge C Khongphatthanayothin A Walsh R Mauleekoonphairoj J Amnueypol M. Common and rare susceptibility genetic variants predisposing to Brugada syndrome in Thailand. Heart Rhythm. (2020) 17:2145–53. 10.1016/j.hrthm.2020.06.027 32619740

[64] Janin A Bessiere F Georgescu T Chanavat V Chevalier P Millat G. TRPM4 mutations to cause autosomal recessive and not autosomal dominant Brugada type 1 syndrome. Eur J Med Genet. (2019) 62:103527. 10.1016/j.ejmg.2018.08.008 30142439

[65] Kyndt F Probst V Potet F Demolombe S Chevallier JC Baro I. Novel SCN5A mutation leading either to isolated cardiac conduction defect or Brugada syndrome in a large French family. Circulation. (2001) 104:3081–6. 10.1161/hc5001.100834 11748104

[66] Tadros R Tan HL Investigators E-N El Mathari S Kors JA Postema PG. Predicting cardiac electrical response to sodium-channel blockade and Brugada syndrome using polygenic risk scores. Eur Heart J. (2019) 40:3097–107. 10.1093/eurheartj/ehz435 31504448 PMC6769824

[67] Parisi P Oliva A Coll Vidal M Partemi S Campuzano O Iglesias A. Coexistence of epilepsy and Brugada syndrome in a family with SCN5A mutation. Epilepsy Res. (2013) 105:415–8. 10.1016/j.eplepsyres.2013.02.024 23538271

[68] Sandorfi G Clemens B Csanadi Z. Electrical storm in the brain and in the heart: epilepsy and Brugada syndrome. Mayo Clin Proc. (2013) 88:1167–73. 10.1016/j.mayocp.2013.06.019 24079686

[69] Veltmann C Barajas-Martinez H Wolpert C Borggrefe M Schimpf R Pfeiffer R. Further insights in the most common scn5a mutation causing overlapping phenotype of long QT syndrome, brugada syndrome, and conduction defect. J Am Heart Assoc. (2016) 5:e003379. 10.1161/JAHA.116.003379 27381756 PMC5015375

[70] Camacho Velasquez JL Rivero Sanz E Velazquez Benito A Mauri Llerda JA. Epilepsy and brugada syndrome. Neurologia. (2017) 32:58–60. 10.1016/j.nrl.2015.03.010 26037409

[71] Peters S Trummel M Denecke S Koehler B. Results of ajmaline testing in patients with arrhythmogenic right ventricular dysplasia-cardiomyopathy. Int J Cardiol. (2004) 95:207–10. 10.1016/j.ijcard.2003.04.032 15193821

[72] Agullo-Pascual E Cerrone M Delmar M. Arrhythmogenic cardiomyopathy and Brugada syndrome: diseases of the connexome. FEBS Lett. (2014) 588:1322–30. 10.1016/j.febslet.2014.02.008 24548564 PMC3989410

[73] Corrado D Zorzi A Cerrone M Rigato I Mongillo M Bauce B. Relationship between arrhythmogenic right ventricular cardiomyopathy and brugada syndrome: new insights from molecular biology and clinical implications. Circ Arrhythm Electrophysiol. (2016) 9:e003631. 10.1161/CIRCEP.115.003631 26987567 PMC4800833

[74] Cerrone M Delmar M. Desmosomes and the sodium channel complex: implications for arrhythmogenic cardiomyopathy and Brugada syndrome. Trends Cardiovasc Med. (2014) 24:184–90. 10.1016/j.tcm.2014.02.001 24656989 PMC4099253

[75] Cerrone M Lin X Zhang M Agullo-Pascual E Pfenniger A Chkourko Gusky H. Missense mutations in plakophilin-2 cause sodium current deficit and associate with a Brugada syndrome phenotype. Circulation. (2014) 129:1092–103. 10.1161/CIRCULATIONAHA.113.003077 24352520 PMC3954430

[76] Scheirlynck E Chivulescu M Lie OH Motoc A Koulalis J De Asmundis C. Worse prognosis in brugada syndrome patients with arrhythmogenic cardiomyopathy features. JACC Clin Electrophysiol. (2020) 6:1353–63. 10.1016/j.jacep.2020.05.026 33121663

[77] Doenst T Nguyen TD Abel ED. Cardiac metabolism in heart failure: implications beyond ATP production. Circ Res. (2013) 113:709–24. 10.1161/CIRCRESAHA.113.300376 23989714 PMC3896379

[78] Aiba T Farinelli F Kostecki G Hesketh GG Edwards D Biswas S. A mutation causing Brugada syndrome identifies a mechanism for altered autonomic and oxidant regulation of cardiac sodium currents. Circ Cardiovasc Genet. (2014) 7:249–56. 10.1161/CIRCGENETICS.113.000480 24795344 PMC4114079

[79] Liu M Sanyal S Gao G Gurung IS Zhu X Gaconnet G. Cardiac Na+ current regulation by pyridine nucleotides. Circ Res. (2009) 105:737–45. 10.1161/CIRCRESAHA.109.197277 19745168 PMC2773656

[80] Liu M Liu H Dudley SCJr. Reactive oxygen species originating from mitochondria regulate the cardiac sodium channel. Circ Res. (2010) 107:967–74. 10.1161/CIRCRESAHA.110.220673 20724705 PMC2955818

[81] Stocchi L Polidori E Potenza L Rocchi MB Calcabrini C Busacca P. Mutational analysis of mitochondrial DNA in Brugada syndrome. Cardiovasc Pathol. (2016) 25:47–54. 10.1016/j.carpath.2015.10.001 26549652

[82] Polidori E Stocchi L Potenza D Cucchiarini L Stocchi V Potenza L. A high number of 'natural' mitochondrial DNA polymorphisms in a symptomatic Brugada syndrome type 1 patient. J Genet. (2020) 99:66. 10.1007/s12041-020-01228-4 32893837

[83] Tafti MF Khatami M Rezaei S Heidari MM Hadadzadeh M. Novel and heteroplasmic mutations in mitochondrial tRNA genes in Brugada syndrome. Cardiol J. (2018) 25:113–9. 10.5603/CJ.a2017.0104 28980288

[84] Zhu HY Wang SW Liu L Chen R Wang L Gong XL. Genetic variants in mitochondrial tRNA genes are associated with essential hypertension in a Chinese Han population. Clin Chim Acta. (2009) 410:64–9. 10.1016/j.cca.2009.09.023 19778529

[85] Sieira J Conte G Ciconte G Chierchia GB Casado-Arroyo R Baltogiannis G. A score model to predict risk of events in patients with Brugada syndrome. Eur Heart J. (2017) 38:1756–63. 10.1093/eurheartj/ehx119 28379344

[86] Sieira J Ciconte G Conte G De Asmundis C Chierchia GB Baltogiannis G. Long-term prognosis of drug-induced Brugada syndrome. Heart Rhythm. (2017) 14:1427–33. 10.1016/j.hrthm.2017.04.044 28479512

[87] Ciconte G Santinelli V Vicedomini G Borrelli V Monasky MM Micaglio E. Non-invasive assessment of the arrhythmogenic substrate in Brugada syndrome using signal-averaged electrocardiogram: clinical implications from a prospective clinical trial. Europace. (2019) 21:1900–10. 10.1093/europace/euz295 31647530

[88] Pappone C Ciconte G Micaglio E Monasky MM. Common modulators of Brugada syndrome phenotype do not affect SCN5A prognostic value. Eur Heart J. (2021) 42:1273–4. 10.1093/eurheartj/ehab071 33595071 PMC8014514

[89] Priori SG Blomstrom-Lundqvist C Mazzanti A Blom N Borggrefe M Camm J. 2015 ESC Guidelines for the management of patients with ventricular arrhythmias and the prevention of sudden cardiac death: The Task Force for the Management of Patients with Ventricular Arrhythmias and the Prevention of Sudden Cardiac Death of the European Society of Cardiology (ESC). Endorsed by: Association for European Paediatric and Congenital Cardiology (AEPC). Eur Heart J. (2015) 36:2793–867. 10.1093/eurheartj/ehv316 26320108

